# Association of cytomegalovirus and other pathogens with frailty and diabetes mellitus, but not with cardiovascular disease and mortality in psycho-geriatric patients; a prospective cohort study

**DOI:** 10.1186/1742-4933-10-30

**Published:** 2013-07-23

**Authors:** Michiel B Haeseker, Evelien Pijpers, Nicole HTM Dukers-Muijrers, Patty Nelemans, Christian JPA Hoebe, Cathrien A Bruggeman, Annelies Verbon, Valère J Goossens

**Affiliations:** 1 Department of Medical Microbiology, Maastricht University Medical Centre, P. Debyelaan 25, PO Box 5800, 6202 AZ Maastricht, the Netherlands; 2 Care and Public Health Research Institute (CAPHRI), PO Box 616, 6200 MD Maastricht, the Netherlands; 3 Department of Internal Medicine, Maastricht University Medical Centre, Maastricht, the Netherlands; 4 Department of Infectious Diseases, Public Health Services South Limburg, South Limburg, Geleen, the Netherlands; 5 Department of Epidemiology, Maastricht University Medical Centre, Maastricht, the Netherlands; 6 Department of Internal Medicine, Erasmus Medical Centre, Rotterdam, the Netherlands

**Keywords:** Herpes viruses, Cytomegalovirus, Frailty, Diabetes mellitus, Morbidity, Mortality

## Abstract

**Background:**

Studies about associations of infections with herpes viruses and other pathogens, such as *Chlamydia pneumoniae* (CP) and *Helicobacter pylori* (HP) with cardiovascular disease (CVD), diabetes mellitus (DM), frailty and/or mortality are conflicting. Since high levels of antibodies against these pathogens occur in the elderly, the role of these pathogens in morbidity and mortality of vulnerable elderly was explored.

**Results:**

Blood samples of 295 community dwelling psycho-geriatric patients were tested for IgG antibodies to herpes simplex virus type 1 and 2, varicella zoster virus, Epstein Barr virus (EBV), cytomegalovirus (CMV), human herpes virus type 6 (HHV6), CP and HP. Frailty was defined with an easy-to-use previously described frailty risk score. Relative risks (RR) with 95% confidence intervals were calculated to evaluate associations between CVD, DM, frailty and pathogens. Pathogens as a predictor for subsequent mortality were tested using Kaplan Meier analyses and Cox proportional hazard models. The mean age was 78 (SD: 6.7) years, 20% died, 44% were defined as frail, 20% had DM and 49% had CVD. Presence of CMV antibody titers was associated with frailty, as shown by using both qualitative and quantitative tests, RR ratio 1.4 (95% CI: 1.003-2.16) and RR ratio 1.5 (95% CI: 1.06-2.30), respectively. High IgG antibody titers of HHV6 and EBV were associated with DM, RR ratio 3.3 (95% CI: 1.57-6.49). None of the single or combined pathogens were significantly associated with mortality and/or CVD.

**Conclusions:**

Prior CMV infection is associated with frailty, which could be in line with the concept that CMV might have an important role in immunosenescence, while high IgG titers of HHV6 and EBV are associated with DM. No association between a high pathogen burden and morbidity and/or mortality could be demonstrated.

## Background

Age related frailty and chronic diseases, such as diabetes mellitus (DM) and cardiovascular disease (CVD) will increase in developed countries, as the number of elderly people in the developed countries is increasing. A quarter of the population in most European countries will be above 65 years in the near future [1]. The pathophysiological mechanisms of ageing and frailty and the exact causal mechanisms of DM and CVD are not completely understood. Chronic inflammation and prior infections with herpes viruses, *Chlamydia pneumonia* (CP) and *Helicobacter pylori* (HP) are suggested to play a role in ageing, frailty, DM and CVD [2-7].

Ageing is associated with deleterious changes in the immune system called immune senescence which have been observed in all mammals studies thus far [8]. Persistent cytomegalovirus (CMV) infections, but no other herpes virus infections have been found to increase immunosenescence [8]. For instance, progressive accumulation of CD 8+ T-cells is correlated with seropositivity to CMV infection [9-12]. It has been hypothesized that accumulation of CD8+ T-cells against one virus reduces the immunity to other pathogens [13,14] which is illustrated by the association of CMV seropositivity with non-responsiveness to anti-influenza vaccination [15]. Furthermore, chronic stimulation of the immune system by CMV may lead to an increasing prevalence of senescent dysfunctional T cells thereby contributing to mortality and frailty [5,14]. Also, Epstein-Barr virus (EBV) has been associated with a remarkable accumulation of EBV specific CD8 T-cells of up to 30% [13,14].

Some seroepidemiological studies have shown associations between infections with herpes viruses and other common pathogens such as CP and HP on one side and on the other side DM, atherosclerosis, dementia, cognitive impairment, and frailty [2-7]. The exact causal mechanisms are not yet known, although chronic inflammation, which is a risk factor for DM type 2 [16], has been hypothesized to be caused by the presence of persistent infection. Indeed, CMV, varicella zoster virus (VZV) and herpes simplex virus (HSV) have been associated with the occurrence of DM in some studies [6,17-19], but other studies could not confirm this association with DM [20]. Similarly, herpes viruses, HP and CP infections have been associated with atherosclerosis and CVD [2,7], whereas others could not confirm these findings [21,22].

Overall, the concept of accelerating immunosenescence by persistent infections and subsequent increased mortality and morbidity has been suggested in some studies. The aim of this study is to determine associations between frailty, CVD, DM and mortality with single pathogens and combined pathogen burden among prospectively followed community dwelling psycho-geriatric patients.

## Results

### Pathogens and morbidity

A total of 295 blood samples were collected from community dwelling psycho-geriatric patients between October 2002 and December 2005. The mean age of these patients was 78.0 years ± 6.7 years and 64% (n = 188) were female. The median follow-up period of the surviving patients was 25 months (range: 12–36 months). Forty four percent (n = 131/295) of the patients were considered frail, 20% (n = 59/295) had DM (all patients had DM type 2) and 48% (n = 143/295) with CVD. Twenty percent of the 295 patients (n = 60) died during the study period of 36 months. Frequencies for single pathogens and pathogen burden are shown in Table 1. VZV and EBV seronegative patients were very small, only 3 and 4 patients respectively. Additionally, 46% (n = 136) were positive for all herpes viruses tested.

**Table 1:** Frequencies for pathogens and the associations between the different pathogens, cardiovascular disease (CVD), diabetes mellitus (DM) and frailty

**Serology test**	**Range of test**	**Median (IQR)**	**Total**	**DM+**	**CVD+**	**Frail+**
	**(U/ml)**	**Titer (U/ml)**	**number(%)**	**(n = 59)**	**(n = 143)**	**(n = 131)**
**HSV**^a^**IgG positive**	30-2000	437 (389–484)	275 (93%)	57 (97%)	133 (93%)	122 (93%)
**HSV**^a^**IgG negative**		-	20 (7%)	2 (3%)	10 (7%)	9 (7%)
**VZV**^a^**IgG positive**	100-2000	994 (612–1403)	292 (99%)	59 (100%)	141 (99%)	128 (98%)
**VZV**^a^**IgG negative**		-	3 (1%)	0 (0%)	2 (1%)	3 (2%)
**EBV**^a^**IgG positive**	11-200	200 (130–200)	291 (99)%	58 (98%)	141 (99%)	128 (98%)
**EBV**^a^**IgG negative**		-	4 (1%)	1 (2%)	2 (1%)	3 (2%)
**CMV**^a^**IgG positive**			226 (77%)	46 (78%)	115 (80%)	108 (82%)^b^
• **AxSYM**	15-250	250 (192–250)				
• **DSX**^**TM**^	40-2000	373 (290–463)				
**CMV**^a^**IgG negative**		-	69 (23%)	13 (22%)	28 (20%)	23 (18%)^b^
**HHV6**^a^**IgG positive**	>1.1	1.67 (1.37-2.34)	193 (65%)	45 (76%)^c^	97 (68%)	90 (69%)
**HHV6**^a^**IgG negative**		**-**	102 (35%)	14 (24%)^c^	46 (32%)	41 (31%)
**CP**^a^**IgG positive**	45-270	81 (59–116)	150 (51%)	28 (47%)	74 (52%)	68 (52%)
**CP**^a^**IgG negative**		-	144 (49%)	31 (53%)	69 (48%)	63 (48%)
**HP**^a^**IgG positive**	20-650	97 (36–211)	173 (59%)	32 (54%)	82 (47%)	77 (59%)
**HP**^a^**IgG negative**		-	121 (41%)	27 (46%)	61 (43%)	54 (41%)
**Pathogen burden**^**d**^		-				
**● Pathogen burden ≤ 4**			53 (18%)	9 (15%)	25 (18%)	18 (14%)
**● Pathogen burden = 5**		-	95 (32%)	18 (31%)	46 (32%)	44 (33%)
**● Pathogen burden = 6**		-	103 (35%)	23 (39%)	46 (32%)	48 (37%)
**● Pathogen burden = 7**		-	44 (15%)	9 (15%)	26 (18%)	21 (16%)

^a^*HSV*: herpes simplex virus, *VZV*: varicella zoster virus, *CMV*: cytomegalovirus, *EBV*: Epstein-Barr virus, *HHV6*: human herpes virus type 6, *CP*: *Chlamydia pneumonia*, *HP*: *Helicobacter pylori*.

^b^RR 1.4 (95% CI: 1.003-2.16).

^c^RR 1.7 (95% CI: 0.96-3.13).

^d^Pathogen burden: number of IgG seropositive pathogens (HSV, VZV, EBV, CMV, HHV6, CP, HP).

None of the single pathogens were significantly associated with CVD, but an association between seropositivity for CMV and frailty (Tables 1 and 2) and association between high human herpes virus type 6 (HHV6) and high EBV antibody titers and DM was observed (Table 3). Neither the total herpes burden (data not shown), nor the pathogen burden (Table 1) was significantly associated with CVD, DM and/or frailty. Additionally, CMV avidity was not associated with CVD or DM.

**Table 2:** **Association between frailty and cytomegalovirus (CMV) IgG titer (AxSYM and DSX**^**™**^**)**

**Test**	**CMV result**	**N**	**Frailty +**	**Frailty –**	**RR (95% CI)**
			**(n = 131)**	**(n = 164)**	
**AxSYM**	CMV titer: <15 AU/ml	69	23 (17.5%)	46 (28.4%)	
	CMV titer: 15–192 AU/ml	55	22 (17.0%)	33 (20.4%)	1.2 (0.71-2.00)
	CMV titer: ≥192 AU/ml	169	86 (65.5%)	83 (51.2%)	1.5 (1.06-2.30)
**DSX**^**™**^	CMV titer: <40 U/ml	67	22 (16.8%)	45 (27.44%)	
	CMV titer: 40–290 U/ml	56	22 (16.8%)	34 (20.73%)	1.2 (0.72-2.03)
	CMV titer: ≥290 U/ml	172	87 (66.4%)	85 (51.83%)	1.5 (1.06-2.35)

**Table 3:** Association between diabetes mellitus (DM) and antibody titers to human herpes virus (HHV6) and epstein-barr virus (EBV)

**Test result**	**N**	**DM +**	**DM –**	**RR ratio (95% CI)**
		**(n = 59)**	**(n = 236)**	
**HHV6**				
• HHV6 titer: <1.1	103	14 (23.7%)	89 (37.7%)	
• HHV6 titer: 1.1-2.3	144	27 (45.8%)	117 (49.6%)	1.38 (0.74-2.66)
• HHV6 titer: >2.3	48	18 (30.5%)	30 (12.7%)	2.76 (1.42-5.36)
**EBV**				
• EBV titer: 0–200 U/ml	144	21 (35.6%)	123 (52.1%)	
• EBV titer: ≥200 U/ml	151	38 (64.4%)	113 (47.9%)	1.73 (1.04-2.91)
**HHV6 and EBV**				
• HHV6 titer <1.1 and EBV titer 0-200	127	15 (25.4%)	112 (47.5%)	
• HHV6 titer 1.1-2.3 and EBV titer > =200 or HHV6 titer >2.3 and EBV titer 0-200	137	32 (54.2%)	105 (44.5%)	2.28 (1.1-4.7)
• HHV6 titer >2.3 and EBV titer > =200	31	12 (20.4%)	19 (8.0%)	3.28 (1.57-6.49)

### Pathogens and mortality

Seropositivity of single pathogens was associated with increased relative risks (RR) of mortality, but these results did not reach statistical significance (Table 4). After adjustment for frailty most relative risks decreased. Almost all patients (Table 1) were seropositive for VZV and EBV. Therefore, VZV and EBV were left out Table 4.

**Table 4:** Unadjusted and adjusted mortality hazard ratios for frailty with 95% confidence intervals

	**Unadjusted RR (95% CI)**	**Adjusted for frailty RR (95% CI)**
**HSV**^a^	1.23 (0.38-3.93)	1.24 (0.39-3.96)
**CMV**^b^	1.60 (0.81-3.15)	1.20 (0.61-2.39)
**HHV6**^c^	1.00 (0.59-1.71)	0.88 (0.5-1.49)
**CP**^d^	1.07 (0.64-1.77)	1.06 (0.64-1.76)
**HP**^e^	1.26 (0.75-2.13)	1.24 (0.73-2.09)

^a^*HSV*: herpes simplex virus.

^b^*CMV*: cytomegalovirus.

^c^*HHV6*: human herpes virus type 6.

^d^*CP*: *Chlamydia pneumonia.*

^e^*HP*: *Helicobacter pylori.*

### CMV seropositivity and frailty

Of 295 patients, 67 were CMV IgG negative both by AxSYM and DSX™. Two hundred and twenty six patients were CMV IgG positive both by AxSYM and DSX™. Discordance was limited to 2 weakly positive results for DSX™ (44.20 U/ml and 49.10 U/ml) and negative results for AxSYM (0.80 AU/ml and 2.10 AU/ml), confirmed by negative results with Vidas, low CMV IgG avidity and/or CMV IgM negative results by DSX™ and considered negative.

Overall, 76.6% (n = 226) of the geriatric patients was CMV IgG seropositive. Only 1 patient was CMV IgM seropositive with low value. CMV was associated with frailty, RR 1.4 (95% CI: 1.003-2.16) and the quantitative tests showed that the highest CMV IgG titers were more often found in the frailty group RR 1.5 (95% CI: 1.06-2.30), see Table 2. Mean CMV avidity was high 76.2% (SD = 7.9).

### Herpesviruses and DM

HHV6 IgG was associated with DM RR 1.7 (95% CI: 0.96-3.13). The quantitative tests demonstrated that high HHV6 IgG antibody titers RR 3.8 (95% CI: 1.42-5.36) and high EBV antibody titers RR 1.7 (95% CI: 1.04-2.91) were associated with DM, Table 3. An additional effect is demonstrated when high HHV6 antibody titers and high EBV antibody titers are combined RR 3.3 (95% CI: 1.57-6.49), Table 3. Antibody titers of HSV, VZV, CMV, CP and HP were not associated with DM.

## Discussion

Prior infections with herpes viruses, HP and CP are reported to be associated with mortality and morbidity (i.e. CVD, DM and frailty), although the results of different studies are often conflicting [2-7,20-22]. Here, 8 pathogens were tested both qualitative and quantitative in geriatric patients. No robust association between the pathogen burden and mortality was found, although all single pathogens showed increased relative risks with mortality. No associations were found between single pathogens or combined pathogens and CVD. DM was associated with two herpes viruses, i.e. HHV6 and EBV, and an association was demonstrated between frailty and CMV.

The association between CMV and frailty was also found by Schmaltz et al. [5]. Whereas Schmaltz et al. reported an association between CMV and frailty in 724 community dwelling women [5], we demonstrated this association in both men and women. Furthermore, we have performed at least 2 quantitative tests for CMV IgG, a quantitative test for CMV IgM and a test for CMV avidity in order to obtain a more complete CMV serology status. The highest CMV IgG titers were found in frail older people and suggest a dose–response relation, as elevated IgG titers to CMV among seropositive non-immunocompromised individuals suggest frequent viral reactivations [23]. No significant association between CMV (and other pathogens) and mortality was found. Power may be too low to show effects of CMV on mortality. The causal mechanism of the association between CMV and frailty is not yet clear. The association between CMV and frailty could be in line with the concept that CMV might have an important role in immunosenescence [8]. Understanding the role of CMV in the immunosenescence hypothesis more clearly may elucidate the association between CMV and frailty. Therefore, it would be interesting to investigate alterations in the immune system, such as CD4/CD8 ratio’s, in these frail patients. Taken together, an association between CMV and frailty was found.

A recent study showed that in the above 85 year olds CMV seropositivity was higher in patients with DM [19], unfortunately no other pathogens were determined in that study. In our study the CMV seropositivity was only slightly higher in patients with DM. However, DM type 2 was related to the presence of high EBV IgG titers and high HHV6 response. Two case reports have described the development of a type 1 DM with HHV6 reactivation [24,25] and two other case reports during an acute EBV infection [26,27]. Reactivations of both viruses could play a role in DM and in the chronic systemic inflammation in DM [3,6,24,25], since high EBV IgG titers and a high HHV6 response suggests reactivation [28,29]. It is unlikely that the presence of DM has resulted in increased anti-HHV6 or EBV antibody titers since anti-viral antibody levels are decreased rather than increased in patients with DM [18,30]. However, little is known about possible causative mechanisms and more evidence is needed to support this association.

One of the strengths of this study is the large number of pathogens that have been studied in this well defined prospectively followed elderly people group. High antibody titers, together with a high CMV avidity in all CMV positive patients indicate non-primary past or recurrent infection [31]. Antibody titers have been useful to study the dose–response relation. In future research measuring a combination of humoral and cellular immunity markers and persisting viral replication and viral gene expression might be valuable for revealing the role of herpes viruses in mortality and morbidity, especially frailty and DM. Understanding the role of CMV in the immunosenescence hypothesis more clearly may elucidate the association between CMV and frailty.

## Conclusion

Of the 8 pathogens studied in this community dwelling psycho-geriatric patients associations have been shown between prior CMV infection and frailty, which could be in line with the concept that CMV might have an important role in immunosenescence and an association between DM and prior infection with HHV6 and EBV was demonstrated. We could not demonstrate an association between a high pathogen burden and morbidity and/or mortality.

## Methods

### Study population

Data from a study with community dwelling psycho-geriatric patients attending the Diagnostic Observation Centre for Psycho Geriatric patients of the Maastricht University Medical Centre between October 2002 and December 2005 were used. For all patients the frailty score, the presence of DM and that of CVD was determined, and blood samples were collected when patients were included. Frailty was defined with an easy-to-use previously described frailty risk score [32]. The frailty score was calculated with the following rule: Age (per 10 years) x 4 + male sex x 10 + no partner x 5 + BMI < 18.5 kg/m2 + cardiovascular disease x 4 + ADL process deficit x 7. The mean of the frailty score was 52 (median: 51, range: 28–76). The cut-off for frailty was set on ≥53 points” [32].

DM was defined as a history of DM or an elevated fasting glucose and increased HBA1c. CVD in this study was defined from hypertension to severe CVD. Patients were prospectively followed for 36 months.

This study was approved by the medical ethical committee of the Maastricht University Medical Centre. Written informed consent was obtained from the patient for the publication of this report and any accompanying images.

### Laboratory techniques

The blood samples were stored as plasma at –80°C. All blood samples were tested for immunoglobulin G (IgG) to detect antibodies to HSV type 1 and 2, VZV, EBV (Virion\Serion, Würzberg, Germany), CMV (AxSYM, Abbott laboratories, Abbott Park, USA and Virion\Serion, Würzberg and in case of discordance also Vidas, BioMérieux Marcy l’Etoile, France), HHV6 (Panbio diagnostics, Grenoble, France), HP (Orion Diagnostica, Espoo, Finland) and to CP (Anilabsystems Ltd., Vantaa, Finland). Additionally, immunoglobulin M (IgM) and CMV IgG avidity (Virion\Serion, Würzberg, Germany) were tested. All antibodies were tested with quantitative enzyme immunoassay (EIA) on the DSX™ Fourplate Automated Elisa processing system, Chantilly, USA. HSV IgG was positive if antibodies were between 30 and 2000 U/ml, VZV IgG 100–2000 U/ml, EBV IgG 11–200 U/ml, CMV IgG AxSYM 15–250 AU/ml, CMV IgG Dynex 40–2000 U/ml, HHV6 IgG >1.1, CP IgG 45–270 U/ml and HP IgG 20–650 U/ml. Further classification of the CMV, EBV and HHV6 titers were made in weakly positive and strongly positive based on the interquartile range. For CMV the lower quartile (AxSYM: <192 AU/ml and DSX™: <290 U/ml) was considered weakly positive and the quartiles above 192 AU/ml (AxSYM) and above 290 U/ml (DSX™) as strongly positive. For EBV the quartiles below 200 U/ml were considered weakly positive and the quartiles ≥200 U/ml were considered strongly positive. For HHV6 the highest quartile (≥2.3 U/ml) was considered strongly positive and the quartiles lower than 2.3 U/ml as weakly positive. All tests were performed according to the manufactures’ instructions.

### Statistical analysis

Prevalence rates were calculated for single infection, herpes burden (aggregated seropositivity to IgG antibodies for HSV1-2, VZV, EBV, CMV and HHV6) and pathogen burden (aggregated seropositivity to IgG antibodies for the herpes burden, CP and HP). The associations between disease (CVD, DM) or frailty and seropositivity for pathogens at baseline were expressed as relative risks with 95% confidence intervals. Chi-square tests and analysis of variance (ANOVA) were performed to test for statistical significance. Kaplan Meier survival analysis and Cox proportional hazard models were used to evaluate whether seropositivity for pathogens is associated with higher risk of subsequent mortality. Follow-up time was calculated from the date of inclusion to the date of death or the date of last follow-up (censoring date). Hazard ratios with 95% confidence intervals (unadjusted and adjusted for frailty) were calculated. A *P*-value of <0.05 was considered statistically significant. Analyses were carried out with SPSS 16.0 (SPSS Inc., Chicago, USA).

## Competing interests

The authors declare that they have no competing interests.

## Authors’ contributions

MH, EP, CH, ND and PN carried out the data analysis. CB, VG an AV participated in design of the study. MH, ND, VG, PN and AV drafted the manuscript. All authors have read and approved the final version manuscript.
